# Modeling Susceptibility versus Resistance in Allergic Airway Disease Reveals Regulation by Tec Kinase Itk

**DOI:** 10.1371/journal.pone.0011348

**Published:** 2010-06-28

**Authors:** Nisebita Sahu, J. Luis Morales, Deborah Fowell, Avery August

**Affiliations:** 1 Center for Molecular Immunology and Infectious Disease and Department of Veterinary and Biomedical Sciences, The Pennsylvania State University, University Park, Pennsylvania, United States of America; 2 Department of Biochemistry & Molecular Biology, The Pennsylvania State University, University Park, Pennsylvania, United States of America; 3 Department of Microbiology & Immunology, University of Rochester School of Medicine & Dentistry, Rochester, New York, United States of America; Centre de Recherche Public de la Santé (CRP-Santé), Luxembourg

## Abstract

Murine models of allergic asthma have been used to understand the mechanisms of development and pathology in this disease. In addition, knockout mice have contributed significantly to our understanding of the roles of specific molecules and cytokines in these models. However, results can vary significantly depending on the mouse strain used in the model, and in particularly in understanding the effect of specific knockouts. For example, it can be equivocal as to whether specific gene knockouts affect the susceptibility of the mice to developing the disease, or lead to resistance. Here we used a house dust mite model of allergic airway inflammation to examine the response of two strains of mice (C57BL/6 and BALB/c) which differ in their responses in allergic airway inflammation. We demonstrate an algorithm that can facilitate the understanding of the behavior of these models with regards to susceptibility (to allergic airway inflammation) (*S_aai_*) or resistance (*R_aai_*) in this model. We verify that both C57BL/6 and BALB/c develop disease, but BALB/c mice have higher *S_aai_* for development. We then use this approach to show that the absence of the Tec family kinase Itk, which regulates the production of Th2 cytokines, leads to *R_aai_* in the C57BL/6 background, but decreases *S_aai_* on the BALB/c background. We suggest that the use of such approaches could clarify the behavior of various knockout mice in modeling allergic asthma.

## Introduction

Mouse models have played significant roles in our understanding of the immunological mechanism(s) leading to the development of allergic airway inflammation. However, various strains of mice respond differently in these models, and there are more than a few examples of gene knockouts that display contrasting phenotypes in different backgrounds [1], [2]. This is exemplified by the known differences in behavior between C57BL/6 and BALB/c backgrounds in models of allergic airway inflammation [3]. For example, analysis of IL-5 or T-bet knockouts, or the absence of eosinophils, has revealed differences in responses dependent on whether they are on a C57BL/6 or BALB/c background [4], [5], [6], [7], [8]. As a result, it is currently difficult to evaluate without ambiguity, the roles of specific molecules in this disease.

In theoretical and empirical infectious disease models, the concept of tolerance (*T*) and resistance (*R*) has been developed [9]. This concept states that animals that are infected with a pathogen may vary in their response, which can be one of complete resistance, i.e. animals are resistant to developing symptoms of an infection. Alternatively, animals can vary in tolerance, such that the symptoms that develop after infection can be mild, and such animals would be said to have a high tolerance, while animals that have severe symptoms would be said to have low tolerance [9]. However, these responses do not address whether animals are susceptible, i.e. able to be infected by the pathogen, or the mechanisms behind their responses. In this case, one can surmise that a tolerant animal mobilizes immune mechanisms to blunt the pathological effects of the infection and so has varying degrees of tolerance. By contrast, a resistant animal may either rapidly reduce pathogen infection, or rapidly down modulate immune mediated pathological responses such that they are viewed as resistant (to developing symptoms). In both cases, the mechanisms can be potentially traced to the presence of robust immune responses.

Here we suggest that similar models could be applied to study diseases like allergic airway inflammation, in which the trigger for disease is not necessarily an infectious agent. In this case, we propose that animals that have mild symptoms of the disease have low susceptibility (*S_aai_, S* for susceptibility with subscript *aai* to denote the disease, allergic airway inflammation, being analyzed), while animals with elevated symptoms have high susceptibility. Animals with no symptoms are resistant (*R_aai_, R* for resistant with subscript *aai* to denote the disease being analyzed). In the first case of animals with varying degrees of susceptibility, this may be related to the nature and intensity of the immune mediated pathology, while in the second case, this could be related to either a lack of response, or strong down modulation of the immune mediated pathology. While all susceptible animals develop symptoms, analyzing the degrees of susceptibility could allow for discrimination of responses that is not currently appreciated with simple scoring of susceptible and resistant. Decreasing susceptibility (e.g. by pharmaceutical approaches) may be enough to significantly reduce pathology.

We suggest that strain specific differences in responses in allergic airway inflammation models may be due to differences in degrees of susceptibility (degrees of response), or resistance (lack of response or strong down modulation of the immune mediated pathology). It would also be useful to be able to categorize the effects of gene knockouts based on these two parameters, rather than merely susceptible and resistant, since targeting such genes could be an approach to decrease susceptibility and symptoms. In this case, an animal with high *S_aai_* would develop allergic airway inflammation at low doses of allergen, while one with low *S_aai_* would require higher doses. By contrast, an animal that is resistant (*R_aai_*) would not develop symptoms regardless of allergen dose. However, thresholds for allergen exposure and development of disease would have to be established. Experiments that vary antigenic exposure could be used to determine whether strains or gene knockouts have increased or decreased tolerance, or resistance.

Ovalbumin (OVA) sensitization is a popular model for allergic airway inflammation. This model is well established and results in the development of allergic airway inflammation [10]. OVA is initially administered with the adjuvant aluminum hydroxide (Alum) via intraperitoneal injections. The mice are then challenged with antigen OVA by respiratory exposure which triggers the onset of symptoms of the disease [10]. However, this model has limitations, including the fact that it may not reflect natural routes of allergen exposure. In addition, long term exposure to OVA results in the development of tolerance, thus reducing the flexibility in experiments that seek to address varying antigen concentrations [11], [12], [13], [14]. The house dust mite (HDM) species *Dermatophagoides pteronyssinus* is a perennial indoor allergen and it is known to exacerbate the symptoms of allergic airway inflammation in patients. HDM causes airway inflammation in at least 10 percent of the general population, and it is known to exacerbate asthmatic symptoms in 90 percent of humans [15], [16], [17], [18]. In mouse models, exposure to whole HDM extracts results in the development of allergic airway inflammation, even without prior sensitization (as is required for OVA) [19]. In addition, chronic exposure does not result in the development of tolerance as is typically observed with OVA [19].

The tyrosine kinase Interleukin-2 Inducible T cell kinase (Itk) plays an important role in the development of Th2 type responses [20], [21], [22], [23]. Using mouse models of allergic airway inflammation as models of allergic asthma, we have previously shown that Itk plays an important role in the induction of Th2 and Th17 responses that regulate the development of this disease [24], [25], [26], [27], [28]. On the C57BL/6 background, we have reported that the absence of Itk abrogates the development of various symptoms associated with the model of allergic asthma such as airway hyperresponsiveness, mucous production, lymphocyte infiltration and inflammatory cytokine production [29]. We previously used the well established model allergen, ovalbumin (OVA) in these studies, however, as mentioned previously, there are some limitations to this model.

Here we have used HDM extracts in a model of allergic airway inflammation, along with WT and Itk null mice on both C57BL/6 and BALB/c background, to determine whether mice lacking Itk are resistant or have reduced susceptibility to the development of allergic airway inflammation. We developed a mathematical model to determine resistance versus susceptibility, and show that as previously suggested, C57BL/6 mice exhibit reduced susceptibility to developing allergic airway inflammation compared to BALB/c mice. However, on the C57BL/6 background, the absence of Itk results in resistance, but on the BALB/c background results in reduced susceptibility. Our approach also provides a generalized approach to determining whether the absence of specific genes in mice affect susceptibility to developing allergic airway inflammation.

## Results

### Analysis of airways hyperresponsiveness in C57BL/6 and BALB/c mice in response to HDM extract

Mouse models of allergic asthma have played crucial roles in our understanding of this disease in humans. However, the mouse strains that are used sometimes respond differently to the same stimulus. Due to the availability of multiple knockouts, two of the most common strains used in asthma models are based on the C57BL/6 and BALB/c mice strains. These studies have indicated that BALB/c mice have a more robust development of allergic airway inflammation than the C57BL/6 [2]. The response of these animals to varying doses of allergen may be able to provide information regarding the nature of these responses (e.g. there may be a difference in the doses required to elicit a response in the various strains, or in knockouts). A large majority of studies of mouse models of allergic airways disease utilize the Ovalbumin/Alum model. However, it is difficult to use this approach when examining dose responses since prolonged airway exposure of mice to OVA results in the development of tolerance and a subsequent reduction in airway inflammation [19]. By contrast, exposure of mice to extracts of HDM does not result in the development of tolerance [19]. HDM also has the advantage of being a bona fide allergen to which humans develop allergic and asthmatic responses [30]. We therefore used this allergen to examine the dose responsiveness of C57BL/6 and BALB/c mice in the development of allergic airway inflammation.

**Figure 1:**
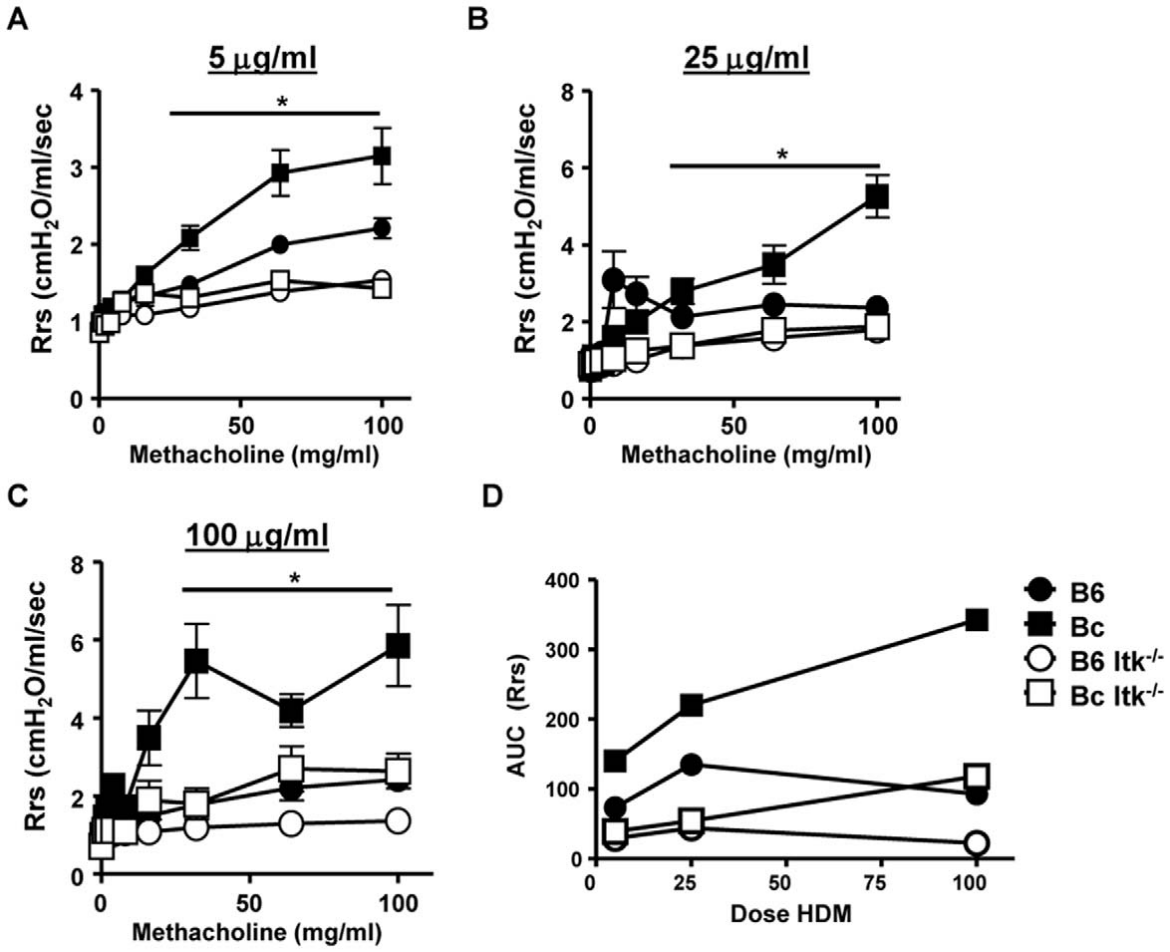
Airways hyperresponsiveness dose response to house dust mite exposure. The indicated strains of mice (B6 = C57BL/6; Bc = BALB/c; B6 Itk^−/−^ =  C57BL/6/Itk^−/−^; Bc Itk^−/−^ =  BALB/c/Itk^−/−^) were exposed to **A**) 5; **B**) 25; and **C**) 100 µg of HDM per day for 10 consecutive days. Twenty four hours after the final exposure, animals were analyzed for hyperresponsiveness to increasing doses of methacholine as described in the materials and methods section (*p<0.05, Bc vs. B6, Bc vs. Bc Itk^−/−^ , B6 vs. B6 Itk^−/−^, Bc Itk^−/−^ vs. B6 Itk^−/−^, 1-way ANOVA). **D**) The Area Under the Curve (AUC) was determined for the methacholine response at each dose of HDM.

We challenged WT C57BL/6 and BALB/c mice with increasing doses (5, 25 and 100 µg/mouse delivered intranasally) of HDM over 10 days. Twenty four hours after the last exposure, we analyzed the mice for airways hyperresponsiveness (AHR). This analysis revealed that WT BALB/c mice had higher AHR than WT C57/Bl/6 mice (**Fig. 1a–c**). However, although both strains had increased AHR responses as the HDM dose increased, there was a limited dose response (**Fig. 1a–c**).

Analyzing the area under the curve (AUC) of the airways resistance measurements provided information as to the dose dependence of these responses. The data (**Fig. 1d**) showed that the BALB/c strain exhibited moderate dose dependence in the AUC for AHR measurements. By contrast, the C57BL/6 mice had little dose response. Analysis of the slopes of the AUC revealed that this was not a good parameter to use for analysis of dose responses, since these slopes were not significantly above 0, suggesting limited dose responsiveness in AHR to HDM exposure.

### Analysis of lung pathology in C57BL/6 and BALB/c mice in response to HDM extract

**Figure 2:**
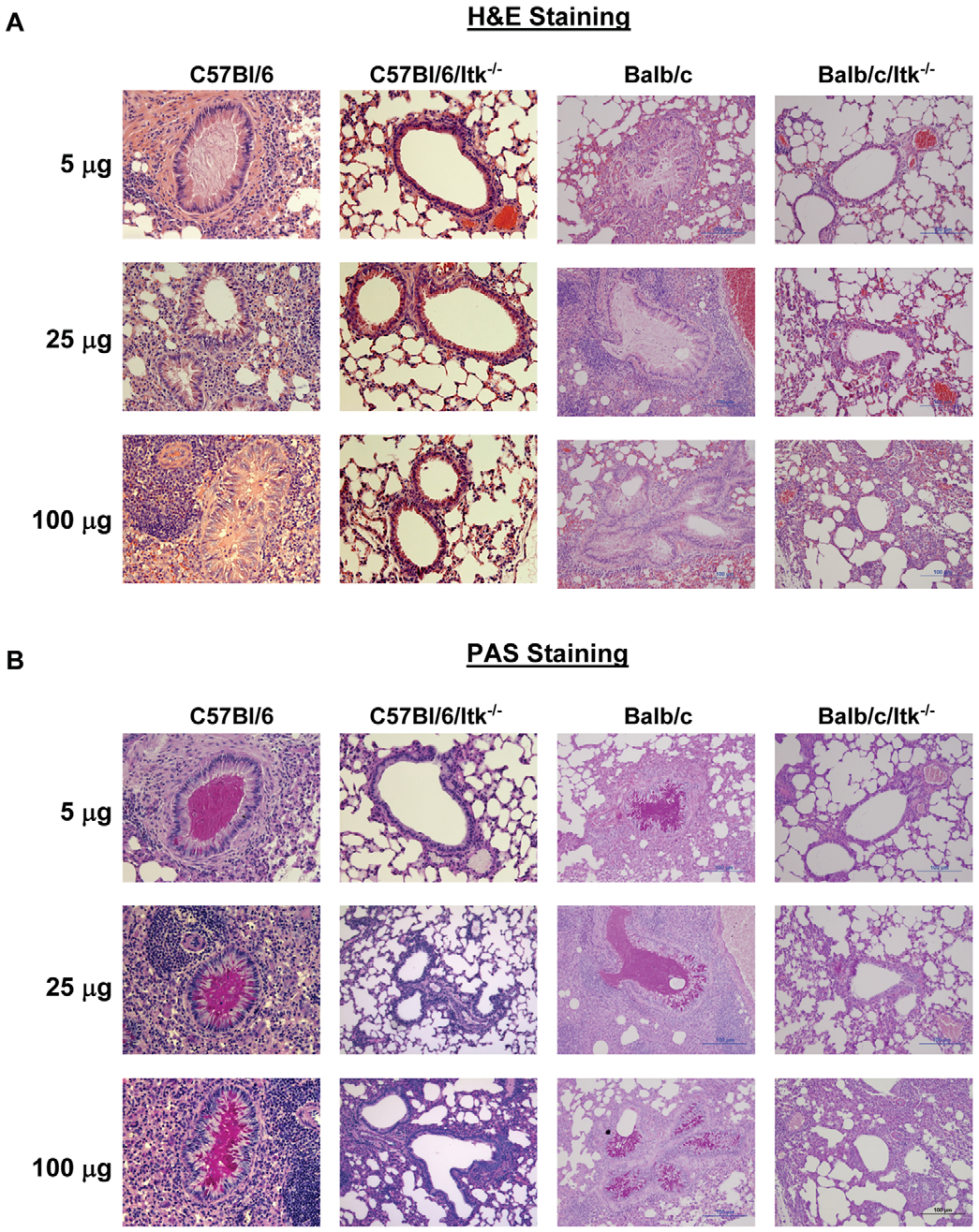
Histopathological analysis of the dose response to house dust mite exposure. The indicated strains of mice were exposed to HDM as in figure 1. Twenty four hours after the final exposure, animals were sacrificed and lungs collected for **A**) H&E staining or **B**) PAS staining. All images are at 10×.

Analysis of lung sections from these mice revealed that both C57BL/6 and BALB/c mice exhibited patterns of lung inflammation and mucous production that reflects the predominant Th2 nature of the response (**Fig. 2**). However, while there was no apparent dose dependency in this pathology in the WT mice, the trend in BALB/c mice suggested an increase in pathology when compared to C57BL/6 mice.

### BALB/c mice have greater Th2 cytokine responses than C57BL/6 mice in a HDM induced model of allergic airway inflammation

**Figure 3:**
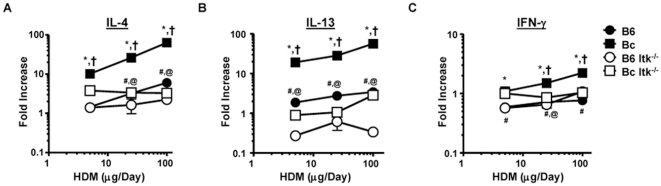
Airway cytokine dose response to house dust mite exposure. A) The indicated strains of mice were exposed to HDM as in figure 1, then sacrificed 24 hours after the final exposure and lungs collected for mRNA analysis. Q-RT-PCR analysis was performed for **A**) IL-4, **B**) IL-13 and **C**) IFN-γ. Data were normalized to PBS exposed animals and expressed as fold increased over the PBS controls (which was set at 1). (*Note log scales on both y- and x-axes*, *p<0.05, Bc vs. B6, ^†^p<0.05, Bc vs. Bc Itk^−/−^, ^#^B6 vs. B6 Itk^−/−^, ^@^p<0.05, Bc Itk^−/−^ vs. B6 Itk^−/−^, students t-test).

We collected lungs from the HDM exposed C57BL/6 and BALB/c mice and analyzed the expression of the Th2 cytokines IL-5, and -13, and the Th1 cytokine IFN-γ. Figure 3 shows the expression of these cytokines in these two mouse strains. We observed that BALB/c mice generated a robust dose-dependent increase in IL-4 production, while in C57BL/6 mice the response was lower (**Fig. 3a**, *note log scales*). Similar results were observed when we analyzed IL-13 expression (**Fig. 3b**). By contrast, while C57BL/6 mice generated little dose dependent increases in the Th1 cytokine IFN-γ, BALB/c mice generated more robust dose dependent increases in this cytokine (**Fig. 3c**).

**Figure 4:**
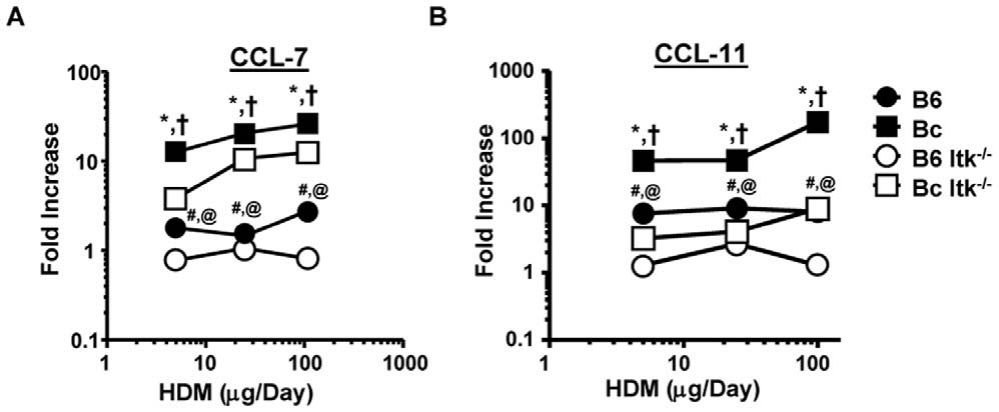
Airway chemokine dose response to house dust mite exposure. A) The indicated strains of mice were exposed to HDM as in figure 1, then sacrificed 24 hours after the final exposure and lungs collected for mRNA analysis. Q-RT-PCR analysis was performed for **A**) CCL-7, **B**) CCL-11 and Data were normalized to PBS exposed animals and expressed as fold increased over the PBS controls as in figure 1. (*Note log scales on both y- and x-axes*, *p<0.05, Bc vs. B6, ^†^p<0.05, Bc vs. Bc Itk^−/−^, ^#^B6 vs. B6 Itk^−/−^, ^@^p<0.05, Bc Itk^−/−^ vs. B6 Itk^−/−^, students t-test).

Analysis of the chemokines CCL7 and CCL11 revealed similar trends, in that WT BALB/c mice had significant dose dependent increases in the expression of these 2 chemokines, although less robust than Th2 cytokine (**Fig. 4**, *note log scales*). These data support the view that BALB/c mice generally exhibit enhanced responses to allergic airway inflammatory stimuli.

### Mice lacking Itk on the C57BL/6 background exhibit reduced responses to HDM induced development of allergic airway inflammation compared to those on the BALB/c background

Mice lacking the Tec family kinase Itk on either C57BL/6 or BALB/c background have T cells that are defective in secretion of Th2 cytokines [20], [21], [22], [23]. We have previously reported that mice lacking Itk on the C57BL/6 background do not develop symptoms of allergic asthma using an OVA model of this disease [27], [29]. Here we have compared mice lacking Itk on both C57BL/6 and BALB/c backgrounds using the HDM dose model described above against their WT counterparts. We have previously reported that in the absence of Itk, C57BL/6 mice do not develop allergic airway disease using the OVA model [26], [27], [29], [31]. Analysis of Itk null mice on both genetic backgrounds revealed reduced AHR responses, and the responses of Itk null mice on a C57BL/6 background were more pronounced than those on the BALB/c background (**Fig. 1a–c**). Itk null mice on both C57BL/6 as well as Balb/c backgrounds exhibited very little lung inflammation, even at the highest concentration of HDM used (100 µg) (**Fig. 2**). As noted for the WT counterparts, there was limited dose responsiveness observed in these parameters.

As previously described in the OVA model, Itk null mice on the C57BL/6 background had significantly reduced cytokine and chemokine responses to HDM compared to WT C57BL/6 mice (**Fig. 3a–c**). In addition, Itk null mice on the BALB/c background had significantly reduced cytokine and chemokine responses to HDM compared to WT BALB/c mice (**Fig. 3a–c**). However, comparing the responses of Itk null mice on the C57BL/6 background to those on the BALB/c background indicated that the Itk null BALB/c mice still exhibited some response to HDM exposure compared to the Itk null C57BL/6 mice (**Fig. 3a–c**). These data support the view that Itk plays a critical role in this response, but the severity varies dependent on the genetic background of the mice.

### C57BL/6 mice have low susceptibility while BALB/c mice have high susceptibility to developing allergic airway inflammation

**Figure 5:**
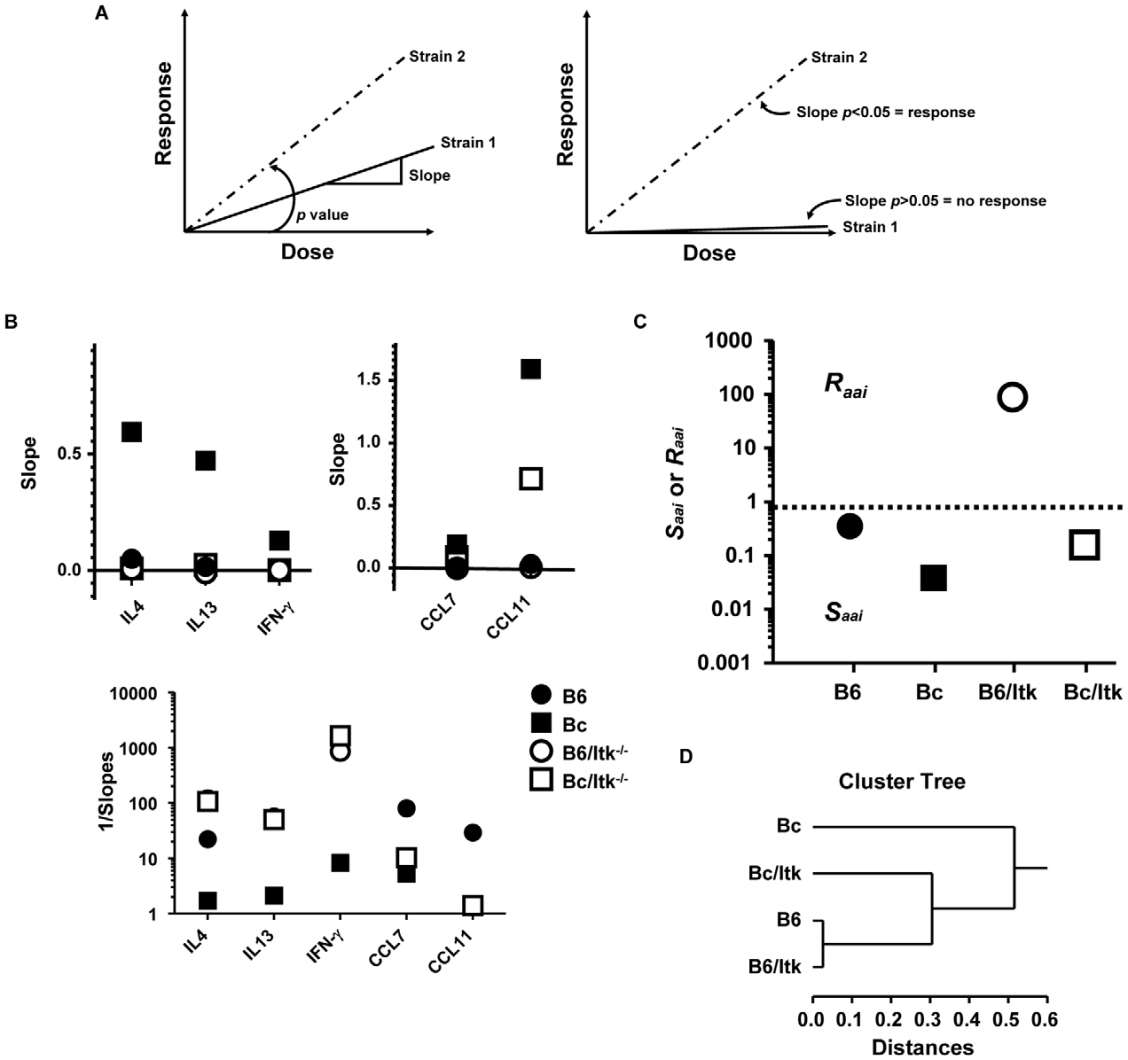
Itk regulates the development of susceptibility or resistance to allergic airway inflammation dependent on background. A) Model of data analysis. Strain 1 and 2 differ with regards to their dose response to allergen. This difference can be captured by the slope of their dose response (Left panel), the significance of which can inform on whether there is a response (*p*<0.05) or not (*p*>0.05) (right panel). These *p* values can be used as described in the text to determine resistance (*R*), i.e. no response (with *p*>0.05,) or the degrees of susceptibility (with *p*<0.05), which is related to the slope of their response, and thus the *p* values of those slopes. **B**) Analysis of the slopes of the IL-4, IL-13 and IFN-γ (left panel) or CCL-7 and CCL-11 (middle panel) dose responses for the indicated mice. Analysis of the inverse of the slopes of the data shown in (A) (right panel, note log scales on the y-axis). **C**) Analysis of Susceptible (*S_aai_*) or Resistance (*R_aai_*) as described in thematerials and methods . The dotted line indicates the cutoff of 0.77 between resistance and susceptibility. **D**) Hierarchical clustering of the values in (C) to generate a cluster tree (B6 = C57BL/6; Bc = BALB/c; B6/Itk^−/−^ =  C57BL/6/Itk^−/−^; Bc/Itk^−/−^ =  BALB/c/Itk^−/−^).

Given these differential responses between C57BL/6 and BALB/c mice, and in the absence of Itk, we attempted to determine whether the absence of Itk led to reduced susceptibility in developing allergic airway inflammation, or resistance. We developed an approach to analyze the response of these mice and place them into categories of varying susceptibility (*S_aai_*) or resistance (*R_aai_*). This approach takes advantage of dose dependent responses in the airways in mice exposed to HDM. The slope of these responses provides a simple parameter that determines whether these animals are responding (i.e. if the slopes are significantly above 0, then there is a dose response and the animals are responding, **Fig. 5a**). The *p* values of these slopes (i.e. whether they are significantly above 0) are collected, transformed (1/average –Log*p*, see materials and methods) and used to determine whether the response of the mice are indicative of degrees of susceptibility (*S_aai_*) or resistance (*R_aai_*). The assumption is that the slopes of the responses to HDM will be significantly higher than 0 if mice are responding (with corresponding decreases in *p* values), regardless of their susceptibility. By contrast, mice that do not respond will have slopes that are not significantly different from 0 (with *p* values >0.05), and so are resistant. The extent to which mice respond is captured by the level of significance of the slopes of their responses (i.e. in the *p* values). A non-biased analysis would reveal mice with varying degrees of susceptibility (*S_aai_*) versus resistant mice (*R_aai_*) using a cutoff of >0.77 for *R_aai_* (1/-Log 0.05, assuming *p* value <0.05 as significant), and <0.77 if mice are *S_aai_*, with values approaching 0.77 interpreted as decreasing susceptibility towards resistance. Thus susceptible animals will have values of <0.77, while resistant mice will have values >0.77.

We rescaled the data shown in figures 3 and 4 to derive simple properties that describe the relationship between the doses of HDM and the cytokine responses of the different strains of mice. Analysis of the slopes of the cytokine and chemokine responses of the WT strains revealed that IL-4 and IL-13 responses in the C57BL/6 mice were much lower than the BALB/c mice (lower slopes, **Fig. 5b**), although both responses had slopes that were statistically significantly above 0 (p<0.05 both strains and cytokines). Analysis of CCL7 responses revealed similar behavior (p<0.05, slopes significantly above 0) (**Fig. 5b**). However, analysis of CCL11 revealed a different behavior, with slopes that were not significantly different from 0 in the two strains of mice. This suggested that CCL11 did not behave in a dose dependent manner that would be useful in determining *S_aai_* or *R_aai_*, (**Fig. 5b**). By contrast, the IFN-γ response had small slopes (reflecting little change versus dose), although the slope was significantly above 0 in the BALB/c mice (p<0.05), but not in C57BL/6 mice, confirming the predominant Th2 nature of this response (**Fig. 5b** **)**.

Combining these data (i.e. the IL-4, -13, CCL7 and 11 responses using the formula described in the materials and methods) revealed that while C57BL/6 mice are not resistant to HDM induced allergic airway inflammation, they have fairly low susceptibility values compared to BALB/c mice, which have high susceptibility values (**Fig. 5c**). Note that inclusion of the IFN-γ response does not change this conclusion, but reduces the separation between the strains.

### Itk regulates susceptibility versus resistance in the development of allergic airway inflammation dependent on the background strain

We next applied these analyses to the strains lacking Itk, and the results were quite revealing. When compared to their WT counterpart, the cytokine and chemokines responses in Itk null mice, on the C57BL/6 background, had slopes that were not significantly above 0 (p>0.05, **Fig. 5a**). By contrast, although the cytokine and chemokine responses in Itk null mice on the BALB/c background had smaller slopes than their WT counterparts, with the exception of IL-4, they were significantly above 0 (p<005 for IL-13, CCL7 and CCL11, **Fig. 5b**). Combining these data resulted in *S_aai_* values supporting a conclusion that on the C57BL/6 background, the absence of Itk results in resistance to HDM induced allergic airway inflammation (**Fig. 5c**). By contrast, on the BALB/c background, the absence of Itk results in reduced susceptibility to HDM induced allergic airway inflammation (**Fig. 5c**). Euclidean hierarchical clustering of these values generates dendrograms that support these conclusions in that the C57BL/6 and C57BL/6/Itk^−/−^ mice clustered close together, the BALB/c/Itk^−/−^ mice clustered close to the C57BL/6, and the BALB/c mice were further apart (**Fig. 5d**). These analyses suggest that on the C57BL/6 background, the absence of Itk results in resistance to the development of HDM induced allergic airway inflammation. By contrast, on the BALB/c background, the absence of Itk reduces the susceptibility of the mice, but they are not resistant.

## Discussion

Mouse models have played significant roles in our understanding of the immunological mechanisms leading to the development of allergic airway inflammation, and are therefore used as models of allergic asthma [2]. Here we compare two commonly used strains in such studies, the C57BL/6 and BALB/c strains, as well as the absence of the Tec family kinase Itk, which regulates effective Th2 responses in models of HDM induced allergic inflammation. Using a new approach to compare whether animals are resistant or have varying degrees of susceptibility to developing allergic airway inflammation, we demonstrate that both strains of mice are sensitive to developing allergic airway inflammation, but that C57BL/6 mice are less susceptible than BALB/c mice. By contrast, we show that in the absence of Itk, C57BL/6 mice become resistant, while on the BALB/c background, these mice have reduced susceptibility, but are not resistant. We suggest that this approach could be used to compare the behavior of various strains of mice and knockouts in allergic inflammation.

We have developed this method based on the model of tolerance and resistance in infectious disease, however, we use susceptibility and resistance (with a subscript that denotes the disease being analyzed (hence *S_aai_* and *R_aai_*). In infectious disease models, the concept of tolerance (*T*) and resistance (*R*) states that animals that are infected with a pathogen may vary in their response, which can be one of complete resistance, i.e. animals are resistant to developing symptoms of an infection or vary in tolerance, such that the symptoms that develop after infection can be severe or mild [9]. We suggest that diseases caused by an immune response, such as allergic airway inflammation, can also be characterized using similar terms. However, in this case, susceptible animals that have mild symptoms of the disease have low susceptibility (*S_aai_*), while animals with elevated symptoms have high susceptibility, and animals that show no symptoms are resistant (*R_aai_*). Importantly, we do not use these terms to suggest a mechanism, but rather the type of response. Furthermore we provide a method to distinguish between susceptible and resistant animals and score their degree of susceptibility to developing symptoms of allergic airway inflammation. When applied to knockout models, this model would allow for the determination of whether a particular gene plays a role in resistance, or decreases symptoms and thus modulates susceptibility. While this could be due to multiple potential mechanisms, pharmaceutical targeting of genes involved in susceptibility may represent better approaches, particularly since they would be able to reduce symptoms (reduce susceptibility), and would perhaps be less likely to have significant effects on other immune responses.

A number of studies have used various approaches to examine strains of mice for their susceptibility to developing airway inflammation [32]. Most of these studies use subjective criteria for determining resistance or susceptibility. However, while resistance is fairly straightforward to identify (e.g. the lack of response), there is not a good way to determine varying degrees of susceptibility. In addition, most studies use allergens at single doses, and it is always possible that at other doses, animals that may seem resistant are actually susceptible, but simply with reduced susceptibility. While a number of studies have compared different strains of mice, and looked at multiple parameters for severity of response (e.g. in [33]), this is usually done using the Ovalbumin model of allergic airway inflammation, which makes it difficult to examine dose responses. A few studies have examined dose responses in the immune response to Ovalbumin, but using different immunizing doses, rather than varying the respiratory exposure. While such analyses are interesting (e.g. immunizing with low doses of ovalbumin in alum predisposes to Th2 responses, while higher doses predispose to Th1 responses [34]), they generally may not represent how humans are sensitized during the development of asthma. The approach used here addresses these concerns by using varying doses of allergen, and combining the responses into one parameter that correlates well with the known behavior of the two strains of mice tested. Validating this approach, we show that dependent on the strain background, absence of the Tec kinase Itk, which regulates Th2 cytokine secretion, either leads to resistance (on a C57BL/6 background) or reduced susceptibility (on a BALB/c background). We suggest that such an approach could also be used in outbred populations including humans, where multiple responses could be combined to provide a single parameter that describes resistance or varying degrees of susceptibility to allergic airway inflammation, or other responses with complex components.

## Materials and Methods

### Mice

WT (C57BL/6 and BALB/c, initially procured from the Jackson Labs) and Itk null mice (on a C57BL/6, kindly provided by Dr. Dan Littman, and BALB/c [35]) were aged between 6–12 weeks old for all these studies. All mice were backcrossed to the respective backgrounds at least 10 generations. All the experiments were approved by the Office of Research Protection's Institutional Animal Care and Use Committee at Pennsylvania State University (protocol # 30752).

### Induction of Experimental Allergic Asthma

We induced allergic airway disease by exposing the mice intranasally to 5, 25, 100 µg of whole HDM extract (Greer Laboratories, Lenoir, NC) resuspended in PBS. Mice were exposed daily for 10 consecutive days, and 24 hours after the final HDM exposure, analyzed for airways hyperresponsiveness, then sacrificed, samples collected for histology, and RNA obtained from lungs as detailed below.

### Analysis of AHR

AHR was analyzed using a flexivent apparatus twenty-four hours after the final HDM challenge. Mice were analyzed for airway hyperresponsiveness using the mechanical ventilator apparatus in response to PBS or increasing doses of aerosolized methacholine from 1 mg/mL to 100 mg/mL as described [29], [36], [37]. Respiratory system resistance (Rrs) values (cm/H_2_0/ml) were plotted vs. methacholine concentration.

### Histology

After AHR analysis, lungs were fixed with 4% formaldehyde and embedded in paraffin wax and cut into thin sections. The sections were then fixed onto slides and stained with Hematoxylin and Eosin (H&E) to examine inflammation, and the level of mucous production determined by using periodic acid-Schiff (PAS) stain.

### Quantitative RT-PCR analysis

Twenty-four hours after the last HDM challenge, total RNA was isolated from lung tissue using Trizol reagent (Invitrogen Life Technologies). Total RNA (5 µg) was reverse transcribed to cDNA with random primers using the Ready-To-Go You-Prime First Strand Beads following manufacturer's instructions (Amersham Biosciences, Piscataway, NJ). PCR was performed in triplicate on an Applied Biosystems ABI PRISM 7300 Sequence Detection System using Taqman Universal PCR master mix with commercially available primers and FAM labeled probes (Assays on Demand™, Applied Biosystems). Relative quantification was performed using the Comparative C_T_ (threshold cycle) method which uses the arithmetic formula 2^−ΔΔCT^ to represent the amount of target gene, normalized to an endogenous reference (GAPDH, in our case) and relative to a calibrator sample. The ΔC_T_ value is determined by subtracting the average GAPDH C_T_ value from the average target gene C_T_ value. The ΔΔC_T_ value is then calculated by subtracting the ΔC_T_ value of the chosen calibrator sample from the ΔC_T_ value of the target gene. The relative gene expression levels were then determined from the equation 2^−ΔΔCT^.

### Data analysis

Statistical evaluation was conducted by using Student's *t* test with a probability value of *P* ≤ 0.05 considered statistically significant. Analysis of the slopes of the dose responses were performed using Graphpad Prizm (note that while the data shown in figure 3 and 4 are log plotted, the slopes were determined using all values). If the *p* values of those slopes are statistically significant, this indicates that the animals responded in a dose dependent fashion since the slopes are significantly non-zero. We then derived the inverse of the average of the −Log_10_ of the *p* values of the slopes (i.e. 1/avg(–Log_10_p(n), where n is the number of responses measured, in this case, the IL-4, IL-13, CCL7 and CCL11 responses). The assumption is that the slopes of the responses to HDM will be significantly higher than 0 if mice are responding (with corresponding decreases in *p* values), even if they have low susceptibility to HDM (and thus respond poorly). By contrast, mice that do not respond will have slopes that are not significantly different from 0 (with *p* values >0.05), and so are resistant. The extent to which mice respond is captured by the level of statistical significance of the slopes of their responses (i.e. in the *p* values). A non-biased analysis would reveal susceptible mice, with varying degrees of susceptibility (*S_aai_*) versus resistant mice (*R_aai_*) using a cutoff of >0.77 for *R* (1/−Log 0.05, assuming *p* value <0.05 as significant) and <0.77 if mice are *S*, with values approaching 0.77 interpreted as decreased susceptibility. Animals were considered susceptible (*S_aai_*) if this value is <0.77 and resistant (*R_aai_*) if this number is >0.77. Generation of slopes, *p* values and statistically analyses were performed using Graphpad Prizm and Microsoft Excel. Hierarchical clustering was performed with SysSTAT (SysSTAT Software) using the same numbers generated as described above (i.e. 1/avg(–Log_10_p(n)).
